# The Role of Electricity in Tabes

**Published:** 1887-03

**Authors:** 


### *THE ROLE OF ELECTRICITY IN TABES.*

One of our esteemed collaborators sends us the following:

"Ziemssen, in his latest edition of his book on electricity in medicine, has, after an experience of thirty years, become a pessimist, contrary to the therapeutic optimism of the specialists. After this the high expectations from the healing powers of electricity will have to be toned down somewhat. This news will hardly affect the remunerative enthusiasm of some electricians, who will, as heretofore, cure tabes and other scleroses by drawing sparks two or more inches long from the astonished and credulous victims."

We may say, in reference to the above, that neither tabes nor any other grave affection of the central nervous system should be combated by electricity alone. It is not the machine or battery, but the medical judgment that directs the conjoint use of electricity and other remedial agencies, that may conquer these maladies; and the medical man who credits the machine alone with the cure, wrongs alike his profession and his patient.

But there remains no doubt in our mind, after long experience and adequate confirmation in the comparative treatment of certain nervous affections with medicine alone, and with conjoint medication and judicious electrization, that results were greatly in favor of the combined plan, especially in epilepsia, paralysis, neuralgia, and sclerosis in all of its forms. The appropriating power for the medicines administered is not only improved by judiciously-selected and prudently-administered electrizations, but favorable molecular changes have been undoubtedly established, from which absolute cures have followed in many of our cases where only such medicines had been used as had previously failed when given without the assistance of conjoint electrization. We have a record of results in our own experience in epilepsy, hemiplegia, and in one or two cases of paraplegia and tabes, sufficiently satisfactory to refute the skepticism of Ziemssen. Electricity, however, is not everything, and it is not the sparks of the static machine (with which we have had comparatively little experience) that does the most good. In fact, two-inch sparks and the shocking effect is rather harmful in tabes than beneficial.

The roller, up and down the spine, especially in the dorso-lumbar area in tabes dorsalis, and to the legs, and the descending constant galvanic current to the head, in cerebral sclerosis, are decidedly beneficial. It is too late in the day of medical progress to ignore them, and it is extremely inconsistent to countenance manual or machinery massage to the extremities in tabes while discountenancing the molecular excitation of the static roller.

